# High-grade chondrosarcoma of the sacrum with mediastinal metastases and a tumor thrombus to the inferior vena cava and right atrium

**DOI:** 10.36416/1806-3756/e20250051

**Published:** 2025-07-22

**Authors:** Flávia Angélica Ferreira Francisco, João Victor Cavalcanti Mesquita Pinto, Edson Marchiori

**Affiliations:** 1. Universidade Federal do Rio de Janeiro, Rio de Janeiro, Brasil.

A 19-year-old female patient complained of shortness of breath and syncope, as well as progressive pain and right leg paresthesia for 6 months. She also developed urinary retention, followed by urinary incontinence, as well as difficulty walking. 

**Figure 1:**
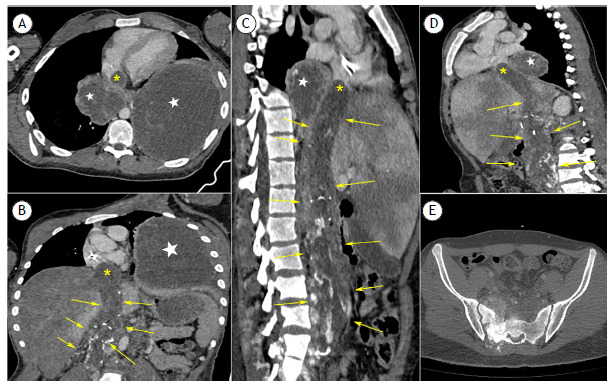
Intravenous contrast-enhanced CT scans of the chest, abdomen, and pelvis, with axial (in A), coronal (in B), and sagittal (in C and D) reconstructions of the thoracoabdominal region, as well as axial CT scan of the pelvis (in E), showing a heterogeneous mass with coarse foci of calcification originating in the pelvic region and extending superiorly through the inferior vena cava (yellow arrows), reaching the right atrium (yellow asterisks). Heterogeneous masses are also present in the right posteroinferior mediastinal region and left lower lobe (in A; white stars), in addition to a mass projecting from the right atrium (yellow asterisks). In E, a mixed lytic and sclerotic lesion is seen on the right side of the sacrum.

CT scans of the chest, abdomen, and lumbosacral spine revealed a mixed lytic and sclerotic infiltrative lesion in the sacrum, with extensive thrombosis in the iliac veins and foci of calcification in between, extending into the inferior vena cava and finally entering the right atrium. Heterogeneous masses with foci of calcification, consistent with metastases, were also present in the right lower mediastinal region and left lower lobe (Figure 1). 

Percutaneous biopsy of the sacral lesion revealed a high-grade chondrosarcoma. The patient underwent pelvic radiation therapy and chemotherapy, but the treatment was discontinued because her clinical condition worsened; she died shortly thereafter. 

Although intravenous leiomyomatosis is the most common cause of neoplastic thrombi extending through the inferior vena cava and reaching the heart, malignant diseases such as leiomyosarcoma, renal carcinoma, adrenal carcinoma, hepatocellular carcinoma, and Wilms tumor can also exhibit this behavior. However, we found no case of a bone tumor showing this particular behavior.^1^ ^-^ ^3^ The case reported herein is of particular interest to pulmonologists because of its clinical presentation (shortness of breath and syncope), as well as the presence of mediastinal metastases. 
